# I don’t see what you’re saying: The maluma/takete effect does not depend on the visual appearance of phonemes as they are articulated

**DOI:** 10.3758/s13423-022-02224-8

**Published:** 2022-12-15

**Authors:** David M. Sidhu, Gabriella Vigliocco

**Affiliations:** 1 Department of Psychology, University College London, London, UK; 2 Department of Psychology, Carleton University, Ottawa, Ontario Canada

**Keywords:** Iconicity, Multimodality, Language processing, Phonology and semantics

## Abstract

In contrast to the principle of arbitrariness, recent work has shown that language can iconically depict referents being talked about. One such example is the maluma/takete effect: an association between certain phonemes (e.g., those in *maluma*) and round shapes, and other phonemes (e.g., those in *takete* and spiky shapes). An open question has been whether this association is crossmodal (arising from phonemes’ sound or kinesthetics) or unimodal (arising from phonemes’ visual appearance). In the latter case, individuals may associate a person’s rounded lips as they pronounce the /u/ in *maluma* with round shapes. We examined this hypothesis by having participants pair nonwords with shapes in either an audio-only condition (they only heard nonwords) or an audiovisual condition (they both heard nonwords and saw them articulated). We found no evidence that seeing nonwords articulated enhanced the maluma/takete effect. In fact, there was evidence that it *decreased* it in some cases. This was confirmed with a Bayesian analysis. These results eliminate a plausible explanation for the maluma/takete effect, as an instance of visual matching. We discuss the alternate possibility that it involves crossmodal associations.

## Introduction

The received view in language sciences has been that language is largely arbitrary (e.g., de Saussure, 1916). However, there is a growing appreciation for the fact that language also has the ability to imagistically *depict* the things to which it refers, in a non-arbitrary relationship termed *iconicity* (Dingemanse et al., 2015; Murgiano et al., 2021; Perniss et al., 2010). This becomes especially apparent when taking a multimodal view of language. For example, gestures allow a person to visually depict an object, while tone of voice allows a person to imitate emotion (for a review, see Murgiano et al., 2021). Beyond this, work has also shown that language sounds themselves have the ability to depict aspects of meaning. Iconicity has been argued to have played a key role in the origin of language (Kendon, 2004; Perniss & Vigliocco, 2014) and to continue to play a role in supporting language development and processing across spoken and signed languages. This is argued to be especially relevant in displaced contexts when language is about absent referents (Motamedi et al., 2022; Murgiano et al., 2021).

**Fig. 1:**
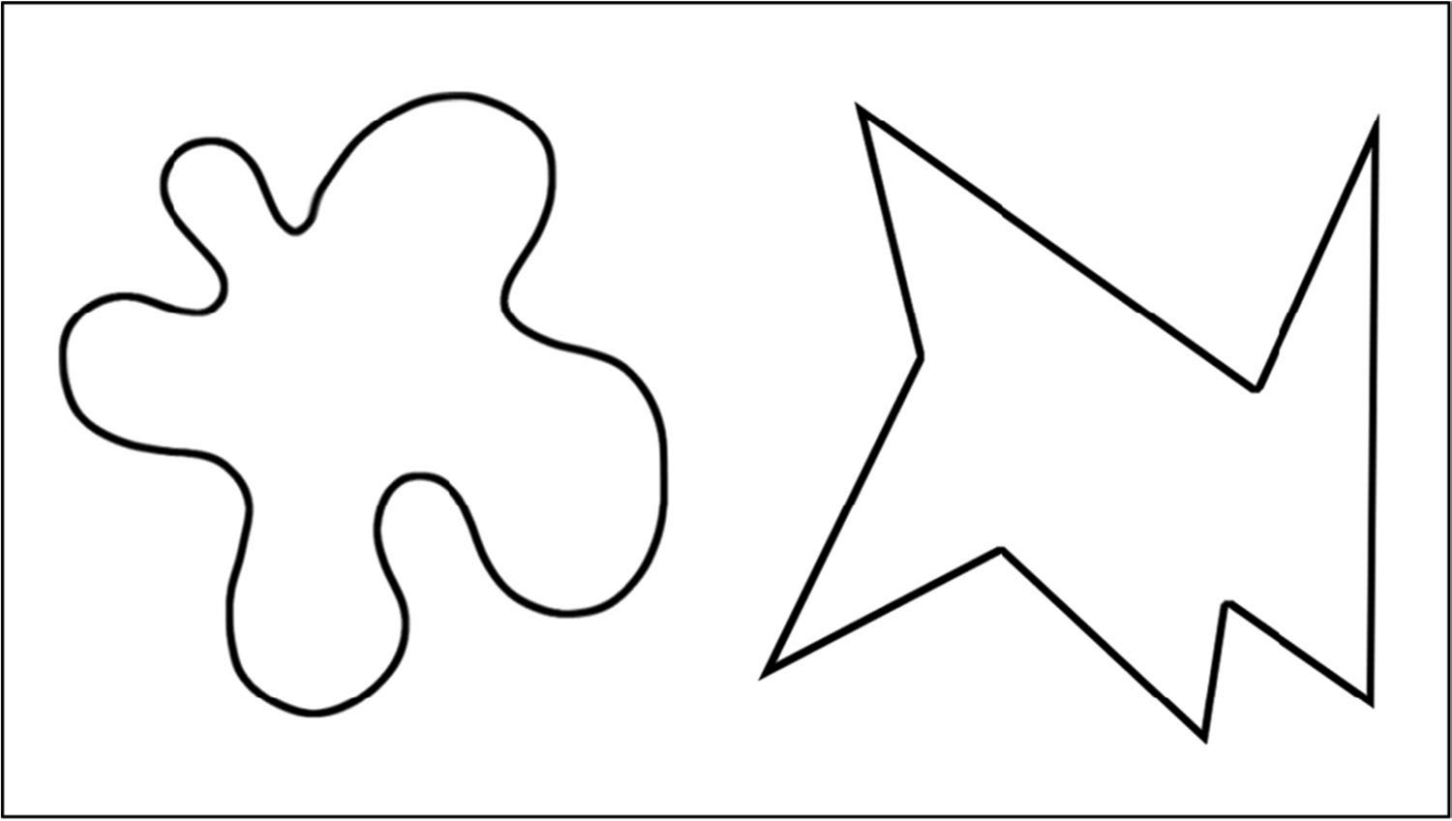
Examples of shapes used in maluma/takete experiments. Most participants pair nonwords like *maluma* with the shape on the left, and nonwords like *takete* with the shape on the right

One example of iconicity in language sounds (also referred to as sound symbolism; see Lockwood & Dingemanse, 2015; Sidhu & Pexman, 2018) is the phenomenon known as the *maluma/takete effect*, referring to an association between certain language sounds (e.g., those in *maluma*) with round shapes, and others (e.g., those in *takete*) with spiky shapes (Köhler, 1929, see Fig. 1). This association emerges on explicit matching tasks (e.g., Ćwiek et al., 2022), implicit tasks (e.g., Parise & Spence, 2012), and on neutral measures (e.g., Asano et al., 2015). However, while the effect has been widely replicated, the question concerning the mechanisms underscoring it, namely *how* these sounds depict visual imagery, is still unanswered. This question is the focus of the current paper.

The maluma/takete effect goes beyond the two eponymous nonwords. In general, sonorants (e.g., /l/, /m/, /n/) are strongly associated with round shapes, while voiceless stops are strongly associated with spiky shapes (e.g., /p/, /t/, /k/; see McCormick et al., 2015; Sidhu et al., 2022). Voiced stops (e.g., /b/, /d/, /g/) are associated with round shapes, but not as much as sonorants. In addition to manner of articulation, *place* of articulation also seems to play a role, with some studies showing that bilabial consonants are associated with round shapes regardless of voicing (D’Onofrio, 2014). In terms of vowels, back vowels (e.g., /ɑ/ as in *law*; /oʊ/ as in *low*) and round vowels (e.g., /oʊ/ but not /ɑ/) are associated with round shapes (D’Onofrio, 2014; McCormick et al., 2015). Because all round vowels are also back vowels in English, these two effects are difficult to disentangle in the current literature. Conversely, high-front vowels (e.g., /i/ as in *Lee*) are associated with spiky shapes (D’Onofrio, 2014; McCormick et al., 2015).

**Fig. 2:**
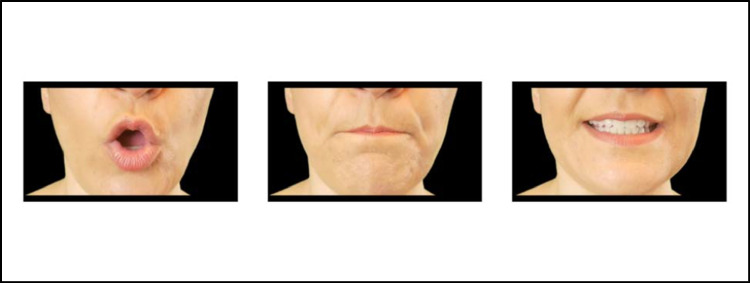
From left to right, examples of the visual appearance of articulations for rounded vowels (/oʊ/), bilabials (/m/) and high-front vowels (/i/)

One possibility is that phonemes depict shapes through direct imitation. That is, the lips could directly imitate the visual appearance of round or spiky shapes. The most obvious instance is the rounding of the lips in the articulation of round vowels (e.g., /u/), which would explain their association with round shapes (see Maurer et al., 2006). Though less immediately obvious, one could also posit that the articulation of bilabials (e.g., /b/) creates a rounded “bulging” appearance of the lips, perhaps also leading to an association with round shapes. Finally, the articulation of high-front vowels involves a lengthening of the mouth that could conceivably be associated with straight lines in spiky shapes. See Fig. 2 for examples of these phonemes being articulated.

Another possibility is that the maluma/takete effect is crossmodal. In this perspective, the sound of phonemes and/or kinesthetic experience of their articulation would serve as perceptuomotor analogies for round or spiky shapes. For example, abrupt changes in amplitude for a phoneme such as /t/ may be analogous to the abrupt changes in outline of spiky shapes (see Lacey et al., 2020). In terms of kinesthetics, consider the burst of air as /t/ is articulated, compared to the more continuous sensations in /m/. This could also be analogous to the abrupt changes in the outline of spiky shapes.

Adjudicating between the different properties of phonemes (i.e., visual, auditory and kinesthetic) has been difficult because they are often confounded. That is, changes in sound come along with changes in kinesthetic experience and visual appearance. This has also made it difficult to settle on a unimodal or a crossmodal explanation. In the present work, we take the approach of highlighting the visual appearance of phonemes (i.e., in accordance with a unimodal explanation), and then measuring the resulting changes in the maluma/takete effect.

In addition, another possibility is that the property linking phonemes and shapes could differ by phoneme. That is, some phonemes may depict shapes unimodally, while others do so crossmodally. For example, the visual similarity between rounded vowels and round shapes is more direct than any between a phoneme and a spiky shape. This could also explain why some studies have found stronger effects for round versus spiky associations (e.g., Flumini et al., 2014; Experiments 1a and 1b when comparing to chance in Sidhu & Pexman, 2015; cf. Nielsen & Rendall, 2011). A recent paper by Cwiek et al. (2022) investigated the maluma/takete effect in speakers of 25 different languages. When examining only the first trial given to participants (to avoid cross-trial comparisons), they observed the maluma/takete effect for round shapes in 22 of the 25 languages, but only in 11 languages for spiky shapes.

Differing mechanisms for round and spiky associations could also explain differences in their development. In a review of the literature, Fort et al. (2018) found that spiky associations increase in infants over time, while round associations do not (showing marginal significance regardless of age). This is consistent with an interpretation in which round associations involve unimodal comparisons, while spiky associations are based on more complicated crossmodal associations (requiring some developmental milestones to be met).

In a pre-registered study, we examined the association between phonemes and either round or spiky shapes, comparing a condition in which participants only heard the nonwords (i.e., audio condition) to a condition in which participants heard and saw each nonword’s articulation (i.e., audiovisual condition). If the associations for rounded vowels, bilabials and front vowels depend on the visual appearance of the lips, then these effects should be larger in the audiovisual condition.

## Method

The preregistration for this experiment can be found at: https://osf.io/nzjsy; data, stimuli and analysis code for this experiment can be found at: https://osf.io/yhpq2/.

### Participants

We collected participants until there were 140 (66 females, 71 males, three not recorded; *M*_age_ = 25.58 years, *SD*_age_ = 8.19) who passed our attention checks and answered a debriefing question saying that their data was appropriate to include (i.e., they had understood and followed the instructions). These participants were recruited using the platform Prolific (https://www.prolific.co/). Sample size was based on a preregistered a priori power analysis, using data from Knoeferle et al. (2017) to estimate effect sizes for our predictors. This power analysis assumed that certain phonemes (i.e., rounded vowels, bilabials and back vowels) would have a 1.5× greater effect in the audiovisual versus the audio-only condition. This power analysis was based on a design in which all participants got 72 trials. When the number of trials was halved, we doubled the sample size to 140.

All participants were fluent in English, and reported normal or corrected-to-normal vision. Participants were compensated at a rate of £7.50 per hour. Two of these participants were removed for having a mean response on our dependent measure (i.e., a rating scale from 1–7) that was greater than three standard deviations from the sample mean (indeed, these participants gave the same response to 31 and 35 of the total 36 trials; thus, this was our exclusion based on careless responding, which was mentioned in the preregistration).

### Materials

Our nonword stimuli consisted of 36 CVCV nonwords. In creating these nonwords we counterbalanced the following factors: manner of articulation (voiceless stops vs. voiced stops vs. sonorants), whether the nonword contained a bilabial consonant or not (always the onset consonant when present), vowel location (back vs. front), and vowel rounding (rounded vs. unrounded). The only exception was that rounded front vowels were not possible with English phonology. There were two items from each combination of factors. See the study’s Open Science Framework (OSF) repository (https://osf.io/yhpq2/) for a list of stimuli.

A professional voice actress recorded a video of each nonword’s articulation. She pronounced each nonword with a neutral intonation, putting the emphasis on the first syllable. These videos made up the stimuli in the audiovisual condition; the audio was extracted from these videos and combined with a black screen for the audio-only condition.

### Procedure

**Fig. 3:**
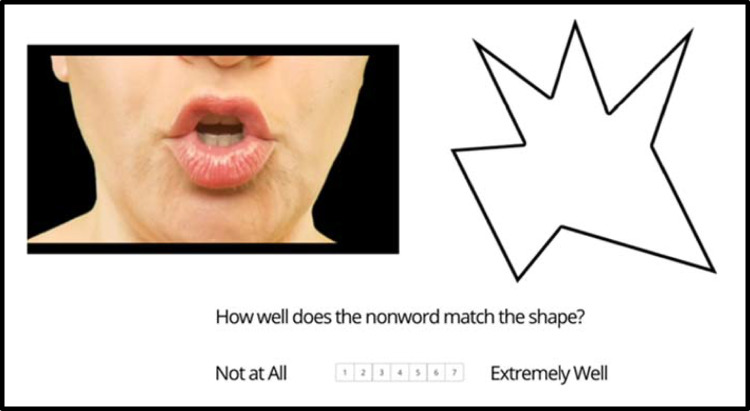
An example trial involving the nonword *boogoe*

Participants were randomly assigned to either an audiovisual or an audio-only condition. On each trial participants were shown either a video of a nonword being articulated (audiovisual condition) or a black screen with only the audio of the nonword’s pronunciation (audio-only condition). They were also shown either a round or a spiky shape, and asked to rate how well the nonword matched the shape from 1 (not at all) to 7 (extremely well); see Fig. 3. Videos were shown along with the shape (i.e., rather than on subsequent screens) to allow participants to make a visual comparison. We judged this to be the most likely condition in which phonemes’ visual features could affect pairings, and thus the most powerful test of the hypothesis that associations depend on visual features.

Each nonword could appear with either a round or a spiky shape, creating 72 potential trials. Each participant was presented with a pseudo-random 36-item subset of these trials. Participants always got 18 trials with a round target, and 18 with a spiky target. Trials were presented in a random order. The experiment was created using the online platform Gorilla (https://gorilla.sc/). All video, audio and visual stimuli can be found at the study’s OSF repository (https://osf.io/yhpq2/).

## Results

Round and spiky trials were analyzed separately. Preregistered confirmatory analyses consisted of linear mixed effects models, conducted using R Statistics (R Core Team, 2021) and the package “lme4” (Bates et al., 2015). We used the package “lmerTest” (Kuznetsova et al., 2017) to generate p values using the Satterwaithe’s degrees of freedom method**.**

### Round trials

**Fig. 4:**
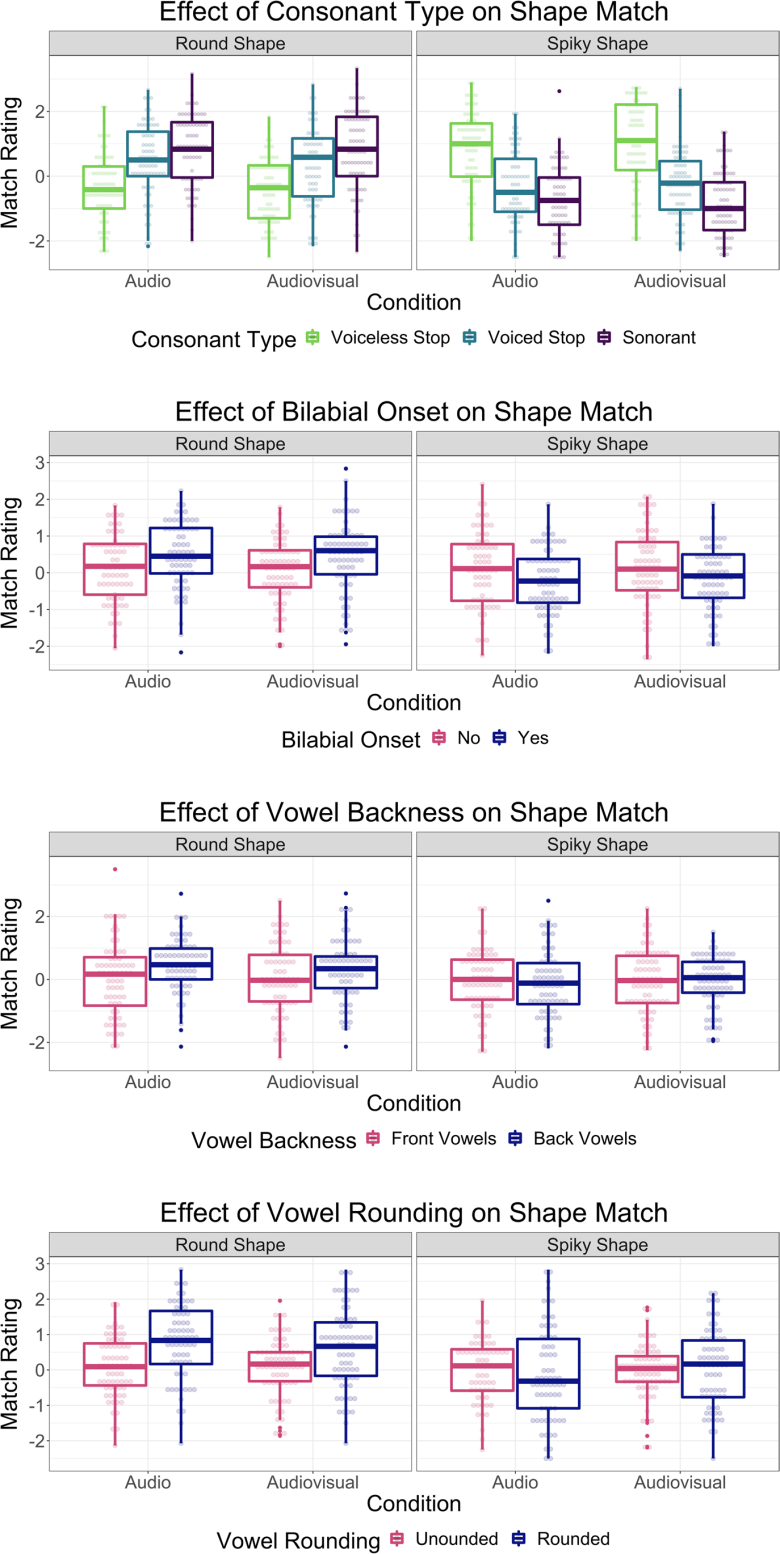
Mean subject ratings of nonword and shape match for each phoneme type. Ratings were made on a 7-point scale, and are shown here with the scale midpoint centered at zero. Dots represent individual subject means. The boxes represent the 25th and 75th percentiles while the horizontal bar inside represents the median. The whiskers extend to the largest value no further than 1.5 times the interquartile range

The dependent variable was the rating of match between the nonword and shape presented on a given trial. The midpoint of this scale was centered at zero in all subsequent reporting to allow interpretation of the intercept. Our predictors were: manner of articulation (dummy coded with voiceless stops as the reference category), bilabial onset (yes vs. no), vowel location (back vs. front), vowel rounding (rounded vs. unrounded) and condition (audiovisual vs. audio; each of these dichotomous predictors were effects coded [0.5] and [-0.5]). We also included an interaction between bilabial onset and condition, and vowel rounding and condition. We began with the maximally complex random effects structure (Barr et al., 2013). This led to a singular fit, which was remedied by iteratively removing the random slope with the lowest associated variance (i.e., the random subject slope for bilabial onset, and random item slope for condition), until the fit was no longer singular. The resulting model is shown in Table 1. This model revealed that relative to voiceless stops, both sonorants (*b* = 1.17, *p* < .001) and voiced stops (*b =* 0.81, *p* < .001) were judged as going along better with round shapes. In addition, nonwords beginning with bilabials (*b* = 0.38, *p* < .001) and those containing rounded vowels (*b* = 0.54, *p* < .001) were judged as better matches for round shapes. Importantly, condition did not interact with the effect of bilabials (*p* = .91) nor rounded vowels (*p* = .26).^1^ See left panels of Fig. 4.

**Table 1:** Results of the mixed effects linear regression predicting the match rating of nonwords and round shapes

Fixed effect	B	SE		t	p	
Intercept	-0.319	0.120		-2.666	.01*	
Manner (Sonorant)	1.174	0.155		7.572	<.001***	
Manner (Voiced Stop)	0.814	0.151		5.401	<.001***	
Bilabial Onset	0.378	0.101		3.757	<.001***	
Back Vowel	0.113	0.142		0.791	.433	
Rounded Vowel	0.536	0.130		4.113	<.001***	
Audiovisual Condition	-0.130	0.138		-0.944	.347	
Bilabial Onset × Audiovisual Condition	0.014	0.118		0.117	.907	
Rounded Vowels × Audiovisual Condition	-0.171	0.150		-1.139	.257	
Random Slope		Variance	r			
Subject Intercept		0.755				
Manner (Sonorant) Slope		1.249	-0.510			
Manner (Voiced Stop) Slope		1.076	-0.520	0.630		
Back Vowels Slope		0.598	-0.310	0.110	0.540	
Rounded Vowels Slope		0.221	0.300	-0.270	-0.060	-0.200
Item Intercept		0.059				

*** *p* < .001, ** *p* < .01, * *p* < .05. Bilabial onset is in comparison to no bilabial onset; back vowel is in comparison to front vowel, rounded vowel is in comparison to no rounded vowel, audiovisual condition is in comparison to the visual condition

In order to quantify the evidence in favour of the null hypothesis, we conducted an exploratory version of the above analysis using Bayesian mixed effects regression via the “rstanarm” package in R (Goodrich et al., 2020). A full introduction to this approach is beyond the scope of this paper and we would refer the reader to Vasishth et al. (2018). Here we conducted a region of practical equivalence (*ROPE*) analysis (see Kruschke, 2018) via the “bayestestR” package in R (Makowski et al., 2019). This method examines the percentage of the highest density interval (*HDI*; i.e., the interval of credible values for each parameter) that falls within a *region of practical equivalence*. That is, instead of treating the null hypothesis as a point value of 0, it treats a range of values around 0, that are practically equivalent to 0, as representing the null. Here we adopt Kruschke’s (2018) suggestion of a range of ± 0.1 standard deviations in the dependent variable as the ROPE. This equates to what Cohen (1988) considered a negligible effect size. In other words, this analysis asks: taking a range of values that we are 95% sure contain the true value of a coefficient, what percentage are practically equivalent to 0. This analysis can also allow one to examine what percentage of the HDI falls on *either side* of 0. That is, of the credible values for a given parameter, how many are positive versus negative.

We simplified the random effects of the model to deal with divergent transitions, and so ran a model that only included random intercepts, as well as a random subject slope for vowel rounding. Our analysis found that 88.12% of the HDI for the bilabial onset × condition interaction fell within the ROPE, while 35.43% did so for the vowel rounding × condition interaction. Thus, this analysis supported the null result for the bilabial onset x condition interaction, but was ambivalent with regards to the vowel rounding x condition interaction. However, a comparison of the HDI on either side of 0 indicated that 93.74% of the credible values for the vowel rounding x condition interaction were *negative.* Note that negative values would indicate the effect of vowel rounding got *smaller* in the audiovisual condition. Thus, we can be confident that if an effect does exist, it would not support the hypothesis that audiovidual information increases the maluma/takete effect for rounded vowels.

### Spiky trials

These analyses were the same as those for the round trials, except that it analyzed trials in which a spiky shape was presented. In addition, the interaction that we tested here was between back vowel presence and condition. We removed the random item slope for condition, and the random subject slope for bilabial onset, to deal with a singular fit. The resulting model is shown in Table 2. This model revealed that voiceless stops were judged as better matches for spiky shapes than both sonorants (*b* = -1.66, *p* <.001) and voiced stops (*b* = -1.19, *p* < .001)^2^. In addition, nonwords with a bilabial onset were judged as worse matches for spiky shapes (*b* = -0.22, *p* = .01). Importantly, condition did not interact with the effect of back vowels (*p* = .82).

**Table 2:** Results of the mixed effects linear regression predicting the match rating of nonwords and spiky shapes

Fixed effect	B	SE		t	p	
Intercept	0.928	0.120		7.743	<.001***	
Manner (Sonorant)	-1.662	0.151		-10.978	<.001***	
Manner (Voiced Stop)	-1.190	0.120		-9.911	<.001***	
Bilabial Onset	-0.215	0.079		-2.727	.011*	
Back Vowels	-0.092	0.113		-0.816	.419	
Rounded Vowels	0.006	0.108		0.057	.955	
Audiovisual Condition	-0.055	0.132		-0.417	.677	
Back Vowels × Audiovisual Condition	0.037	0.164		0.227	.820	
Random Slope		Variance	r			
Subject Intercept		1.200				
Manner (Sonorant) Slope		1.857	-0.77			
Manner (Voiced Stop) Slope		0.693	-0.53	0.86		
Back Vowels Slope		0.486	0.00	-0.07	-0.13	
Rounded Vowels Slope		0.319	0.28	-0.04	0.38	-0.39
Item Intercept		0.025				

*** *p* < .001, * *p* < .05. Bilabial onset is in comparison to no bilabial onset; back vowel is in comparison to front vowel, rounded vowel is in comparison to no rounded vowel, audiovisual condition is in comparison to the visual condition

The exploratory Bayesian version of the above analysis included random subject and item intercepts, and a random subject slope for back vowels. It indicated that 77.63% of the HDI for the interaction between back vowels and condition fell within the ROPE.

## Discussion

We investigated the maluma/takete effect, a robust association between certain phonemes and either round or spiky shapes. We asked whether these associations operate unimodally or crossmodally. To that end, we divided participants into an audio-only condition, and an audiovisual condition in which they both heard nonwords and saw their articulation. For both round and spiky shapes, we failed to find any effect of audiovisual condition. There was a non-significant interaction between bilabial onsets and condition (88.12% of HDI in the ROPE) and round vowels and condition (35.43% of HDI in the ROPE) for round shape associations. A follow-up Bayesian analysis determined that if an interaction did exist for vowel rounding, it was likely to go in the opposite direction to that predicted (i.e., a weaker effect of vowel rounding in the audiovisual condition). There was also a non-significant interaction between front (vs. back) vowel presence and condition for spiky associations (77.63% of HDI in the ROPE).

These results suggest that the visual appearance of phonemes’ articulations is not a major contributor to the maluma/takete effect. Instead, we consider other features such as phonemes’ sounds or kinesthetic experiences are likely to be the main contributors to the association. Rounded vowels are associated with a lower second formant (Kawahara, 2021), and this acoustic property could be associated with visual roundness. However, unrounded back vowels also have a lower second formant than front vowels and were not associated with roundness. Thus, this account may not be sufficient. We believe it to be more likely that the kinesthetic experience of rounding the lips when articulating rounded vowels is what is associated with the visual appearance of round shapes. For other associations (i.e., for sonorants, voiced stops, voiceless stops and bilabials), it is more difficult to decide between phonology and kinesthetics.

The main contribution of this work is to exclude a plausible mechanistic account of the maluma/takete effect (in particular for round shapes) as a unimodal phenomenon. This increases the plausibility of the alternative hypothesis, namely that it is a genuine crossmodal effect. In the *Introduction*, we gave examples of imitative gestures and emotional tone of voice as illustrations of iconicity. However, the maluma/takete effect would seem to have more in common with crossmodal examples of iconicity such as prosody. Iconic prosody includes examples such as the use of rising pitch to indicate that something is high in the air, or low pitch to indicate a large object (Motamedi et al., 2022; Perlman & Cain, 2014).

These results have implications for the development of the maluma/takete effect. Studies have shown the maluma/takete effect is observable in 1-year-old infants using both behavioural (e.g., Pejovic & Molnar, 2017) and neural (e.g., Asano et al., 2015) measures (for a review, see Fort et al., 2018). As mentioned in the *Introduction*, Fort et al. (2018) found that spiky associations increased over time while round associations did not. One explanation could have been that round associations involve unimodal comparisons, while spiky associations require cognitive maturation in order to make crossmodal comparisons. However, the present results suggest that this is not the case, and that both round and spiky associations are crossmodal. This may be the reason that the maluma/takete effect is not observable prior to the age of 1, as some amount of cognitive maturation is required for both round and spiky associations (see Pejovic & Molnar, 2017). The earlier appearance of round associations could also have to do with the earlier acquisition of sonorants such as /m/ and /n/ as compared to the voiceless stops /t/ and /k/ (Sander, 1972).

We were also able to quantify the separate associations between several phoneme categories, and round and spiky shapes. One notable observation is that bilabial consonants have an association with round shapes, independent of the manner of articulation (see also D’Onofrio, 2014). This explains the association between /b/ and round shapes observed throughout the literature (e.g., Westbury et al., 2018), which is much stronger than that of other voiced stops. In addition, although vowel rounding and backness are often grouped together in the literature, by modelling them separately, we found that only vowel rounding was associated with round shapes. As noted earlier, there is an obvious kinesthetic/visual link between vowel rounding and round shapes. There is no such obvious link for vowel backness, which may explain its lack of an effect, and argue for a prominence of kinesthetic experience (vs. sound) in the maluma/takete effect.

It is also worth noting that no vowels were associated with spiky shapes. In case the effect would be stronger for /i/ than /eɪ/, we ran a supplementary exploratory analysis exploring the association of front vowels with spiky shapes when only including front vowel nonwords that had /i/ in the first syllable (assuming that this would make /i/ more salient; see Klink & Wu, 2014). Even in this case, vowel backness was not a significant predictor of spikiness (*p* = .61). The discrepancy with existing literature may lie in the fact that many previous studies have used a decision anchored by both a round and a spiky shape, and compared front vowels with back rounded vowels (e.g., Sidhu & Pexman, 2017). Thus, front vowels may be dissociated with roundness when compared to back rounded vowels, but not associated with spikiness in and of themselves (see also Westbury et al., 2018). This further adds to the argument that associations with round and spiky shapes could depend on different mechanisms. Earlier we reviewed several studies in which there was a larger effect for round shapes than spiky ones. Tentatively, we might speculate that this is in part because round associations derive from both consonants and vowels, while spiky associations only derive from consonants.

There are several limitations to this study. One is that because we tested adults who are familiar with the visual appearance of phonemes’ articulations, we cannot rule out the possibility that participants relied on this knowledge even in the audio-only condition. It is also true that the nonwords used in this study were relatively simple (i.e., all CVCV), with consonants and vowels coming from the same category. One might argue that visual features would be relied upon more so for difficult-to-process linguistic stimuli. Indeed, work has shown that mouth movements can be used to process language in particularly difficult contexts (e.g., Krason et al., 2021).

In conclusion, the present study showed that highlighting visual features of articulation does not enhance the canonical maluma/takete effect. This suggests that the effect arises from a crossmodal matching between non-visual features of phonemes, and shapes. This suggests a tight link between linguistic and non-linguistic aspects of communication, a link that could have been exploited during language origin to develop language as a referential system (Perniss & Vigliocco, 2014), and can be used during language development and processing to ground properties of the communication to the properties of referents being talked about.

## Footnotes

1 We ran a supplementary exploratory analysis that included a three-way interaction between bilabials, rounded vowels, and condition, as well as all component two-way interactions. This was to explore the possibility that coarticulation between bilabials and rounded vowels could have increased effects of lip rounding in nonwords containing both. However, the three-way interaction was not significant (p = .55), nor was the interaction between bilabials and rounded vowels (p = .63).

2 We compared sonorants and voiced stops as matches for round and spiky shapes, in a pair of supplementary exploratory analyses. We found that sonorants were rated as better matches for round shapes than voiced stops (*b* = 0.36, *p* = .02). Conversely, voiced stops were rated as better matches for spiky shapes than sonorants (*b* = 0.47, *p* < .001).
